# Stacking Chairs: Local Sense and Global Nonsense

**DOI:** 10.1177/2041669517752372

**Published:** 2018-01-19

**Authors:** Nicholas E. Scott-Samuel, Hiroshi Ashida, P. George Lovell, Tim S. Meese, D. Samuel Schwarzkopf

**Affiliations:** University of Bristol, UK; Kyoto University, Japan; 3041 University of Abertay Dundee, School of Social and Health Sciences, UK; Aston University, School of Life and Health Sciences, Birmingham, UK; 1415 University College London, UK

**Keywords:** 3D perception, depth, perception, illusion

## Abstract

We report a confusing stimulus which demonstrates the power of local interpretation of three-dimensional structure to disrupt a coherent global perception.

Figure 1 shows a photograph of nine stackable chairs, leaning back at an angle against a wall. For all observers (n = 40+, recruited *ad hoc* via Facebook, where the stimulus was displayed), this image elicits confusion. If the number of chairs is reduced below four, the effect disappears.

**Figure 1.**
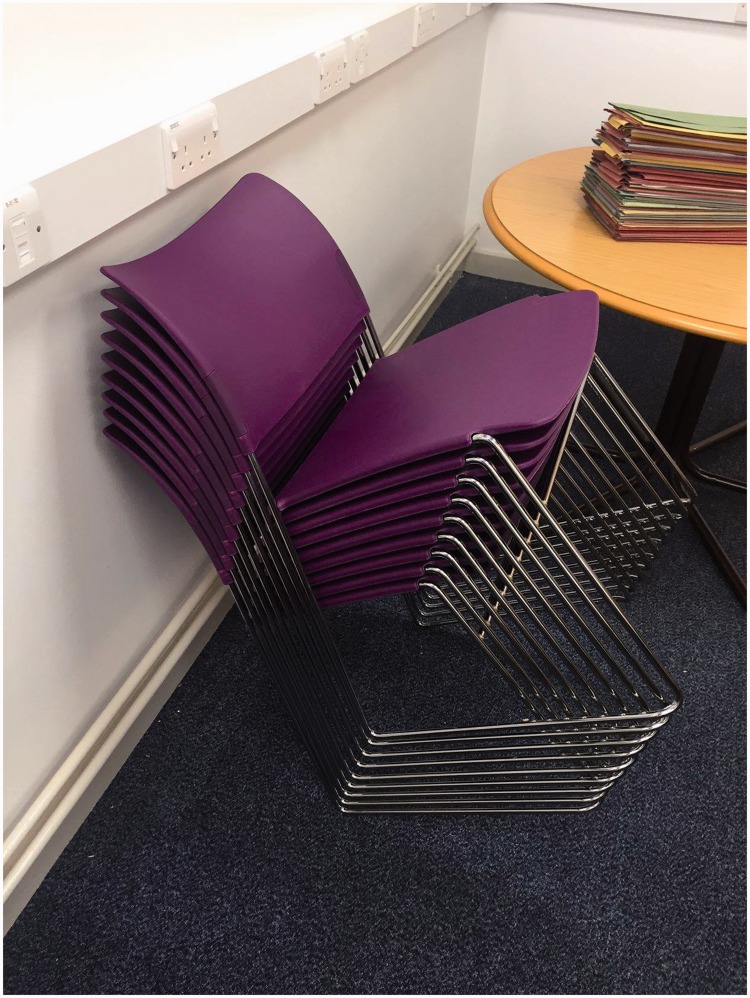
The stacking chairs.

Figure 2(a) is an annotated version of Figure 1. The local interpretation of three-dimensional structure – at each ‘corner’, *i.e.* AD, BC and EF – is generally unambiguous (apart from AD, which flips in depth in a Necker-cube-like manner for some observers). But the repetition of the stacked elements along the virtual contours AD, BC and, to a lesser extent, EF suggests a change in depth along those lines which does not actually exist. Figure 2(b) makes this explicit: an abstracted version of the image reveals an alternative interpretation, which fails to correspond to reality – the repetitive structure now looks more like a stack of quadrilaterals rising from the ground plane.

**Figure 2.**
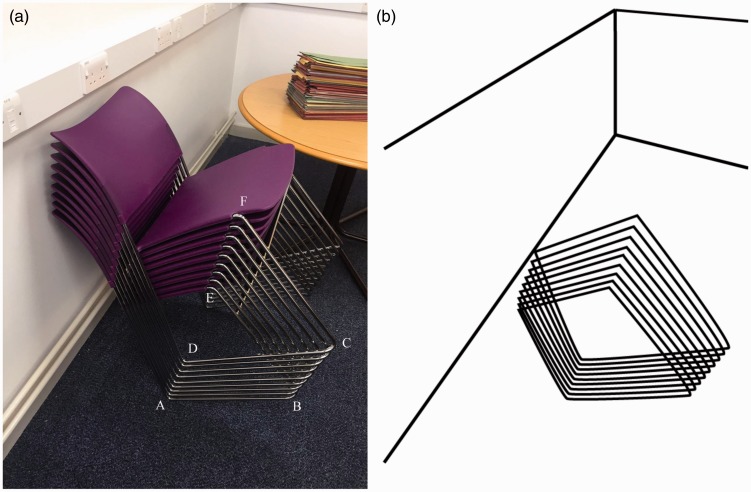
An annotated (a) and an abstracted (b) version.

The stimulus superficially calls to mind the Penrose triangle (Penrose & Penrose, 1958), the critical difference between the two being that the latter has no real-world interpretation (it is a paradoxical figure, and geometrically impossible), whereas the former does: A, B, C, D, E and F all being in roughly in the same vertical plane. But the misleading local three-dimensional form at each corner is so compelling that we see an incoherent Gestalt instead, with the parallel planes ABCD and BCFE not appearing to be such. There is local sense, but global nonsense.

Online Video 1 reveals the true three-dimensional form of the stimulus. Nick Scott-Samuel (in whose office the illusion was observed) reports that it obtains in real life as well as in images, even when sober.

## Supplementary Material

Supplementary material
